# Diabetes compromises tight junction protein claudin 14 in the urinary bladder

**DOI:** 10.1007/s00441-024-03908-4

**Published:** 2024-08-20

**Authors:** Soumitra Mohanty, John Kerr White, Andrea Scheffschick, Berenice Fischer, Anuj Pathak, Jonas Tovi, Claes-Göran Östenson, Pontus Aspenström, Hanna Brauner, Annelie Brauner

**Affiliations:** 1 Department of Microbiology, Tumor and Cell Biology, Karolinska Institutet, Stockholm, Sweden; 2 Division of Clinical Microbiology, Karolinska University Hospital, Stockholm, Sweden; 3 Division of Dermatology and Venereology, Department of Medicine, Solna, Stockholm, Sweden; 4 Capio Health Care Center, Solna, Sweden; 5 Department of Molecular Medicine and Surgery, Karolinska Institutet, Stockholm, Sweden; 6 Rudbeck Laboratory, Department of Immunology, Genetics and Pathology (IGP), Uppsala University, Uppsala, Sweden; 7 Division of Dermatology and Venereology, Karolinska University Hospital, Stockholm, Sweden; 8 Center for Molecular Medicine, Karolinska Institutet, Solna, Sweden; 9 Current Address: Biotechnology Research and Innovation Council-National Institute of Biomedical Genomics (BRIC-NIBMG), Netaji Subhas Sanatorium, Kalyani, India

**Keywords:** Urinary bladder, Diabetes, Epithelial cells

## Abstract

Infections are common in patients with diabetes. Moreover, increasing incidence of antibiotic resistance impedes the complete bacterial clearance and calls for alternative treatment strategies. Along with antibacterial resistance, compromised host conditions create a favorable condition for the disease progression. In particular, cell junction proteins are of major importance as they contribute to a tight cell barrier, protecting against invading pathogens. However, the impact of high glucose on cell junction proteins has received little attention in the urinary bladder but merits closer investigation. Here, we report that during diabetes the expression of cell junction protein, claudin 14 is compromised in the human urine exfoliated cells and in the urinary bladder of type 2 diabetic mouse. Further in vitro analysis confirmed a direct correlation of lower intracellular calcium levels with claudin 14 expression in high glucose-treated human uroepithelial cells. Moreover, external calcium supplementation in high glucose-treated cells significantly affected the cell migration and restored the claudin 14 expression through focal adhesion and β-1 integrins. Strengthening the epithelial barrier is essential, especially in individuals with diabetes where basal calcium levels could contribute.

**Supplementary Information:**

The online version contains supplementary material available at 10.1007/s00441-024-03908-4.

## Background

Tight junctions are primarily located on the apical-lateral surface of epithelial cells involving integral transmembrane proteins linked to cytoskeletal network. Major transmembrane proteins present in the tight junctions include claudins, occludin, and junctional adhesion molecules.

The bladder epithelium is well distributed with tight junction proteins including tight junction protein 1 (TJP1), occludin, and claudin 4, 8, 12, and 14 (Acharya et al. 2004; Mohanty et al. 2020). Tight junction proteins contribute to a tight barrier between the invading pathogens in the lumen and the tissue. Apart from inhibiting pathogen entry, the cell barrier selectively serves the passage of nutrients and electrolytes by forming complex protein-protein interactions.

High glucose has been shown to act differently in different cells/organs. Recent evidence suggests that high glucose detrimentally affects the tight junction protein, occludin in the urinary bladder (Mohanty et al. 2022). Additionally, high glucose is also known to downregulate the expression of tight junction proteins, such as occludin, claudin 5 and TJP1 in neuro-endothelial cells (Schneider et al. 2011). In contrast, high glucose exposure did not cause any noticeable change in the protein expression of occludin, claudin 1 and TJP1 in corneal epithelial cells (Alfuraih et al. 2020). This confirms the multi-faceted effect of high glucose on different cell lineages. Therefore, we evaluated the effect of high glucose on the bladder tight junction proteins claudin 1, 4, 14, TJP1, and E cadherin and the mechanism of action on bladder tight junction protein.

## Methods

### Study participants and collection of samples

The study was approved by the Regional Ethics Committee, Stockholm, and performed in accordance with the Helsinki Declaration (2018/603-32). Informed consent was obtained from all patients and volunteers participating in the study. Individuals with diabetes mellitus (DM) and non-diabetic controls were included, while those with ongoing urinary tract infections (UTI), any antimicrobial treatment or estrogen supplementation, were excluded (Mohanty et al. 2022).

### In vivo mouse model

Mouse experiments were approved by the Northern Stockholm Animal Ethics Committee, and experiments were carried out according to the guidelines of the Federation of Laboratory Animal Science Association and in compliance with the Committee’s requirements (10370-2018). Ten-week-old female db/db (BKS (D)-*Leprdb*/JorlRj) mice with hereditary type-2 diabetes and wild-type C57BL/6j mice were included in the study. All mice were transurethrally infected with uropathogenic *E. coli* CFT073 following standard procedures (Mohanty et al. 2022).

### Cell lines and culture conditions

Telomerase-immortalized human uroepithelial cells (TERT-NHUC) (kindly provided by M. A. Knowles, Leeds, UK) were grown in EpiLife medium supplemented with 1% of human keratinocyte growth supplement (Life technologies) and cultured at 37 °C and 5% CO_2_. Hyperglycemia was created by exposing cells to 30 mM glucose for 24 to 72 h as appropriate. Normoglycemia, 6 mM glucose, was obtained with the culture media. For calcium staining, Fluor-AM (10 nM, Life technologies) was used. Extracellular calcium (CaCl_2_) (Sigma) and EGTA (Sigma) were supplemented at 1 mM. Cellular cytotoxicity after CaCl_2_ treatment was evaluated using standard trypan blue staining.

### Total RNA isolation and real-time PCR

Total RNA was extracted using the RNeasy Mini kit (Qiagen) according to the manufacturer’s protocol, cDNA was synthesized, and real-time PCR for human and mouse specific genes were analyzed using gene specific primers as described earlier (Mohanty et al. 2022). All the primers used in this study are mentioned in Supplementary Table 1. Relative expressions of target genes were presented as the fold-change relative to non-treated controls.

### Immunofluorescence of bladder sections and cells

Bladder tissue and cells were fixed in 4% PFA. For tissue sections, first paraffin blocks were prepared and cut into 4-µm sections followed by deparaffinization, rehydration, and permeabilization before antibody staining. Similarly, TERT-NHUC cells after PFA fixation were permeabilized and stained with primary antibody. Following secondary antibody staining, nucleus was counter stained using DAPI. Antibodies used are mentioned in Supplementary Table 2. Coverslips containing cells and bladder sections were mounted using Fluoromount G (Invitrogen). Imaging was performed with a Zeiss LSM 700 confocal microscope and a Deltavision widefield fluorescence microscope. Fluorescence intensity per unit area was analyzed in ImageJ software.

### Flow cytometry

To investigate the intra cellular protein expression of claudin 14, TERT-NHUC cells were harvested after 36-h glucose treatment. Cells were fixed, permeabilized, and blocked prior to the addition of primary antibody as described earlier (Mohanty et al. 2022). Cells were resuspended in PBS and data acquired on a BD LSRFortessa™ and analyzed in FlowJo software.

### Statistical analysis

All statistical tests were performed in GraphPad Prism version 5. Data differences were obtained from Student’s unpaired *t*-test, paired Student’s *t*-test and non-parametric one-way ANOVA, and Dunnett’s multiple comparison tests as appropriate. Differences with *p* values below 0.05 were considered statistically significant.

## Results and discussions

**Fig. 1:**
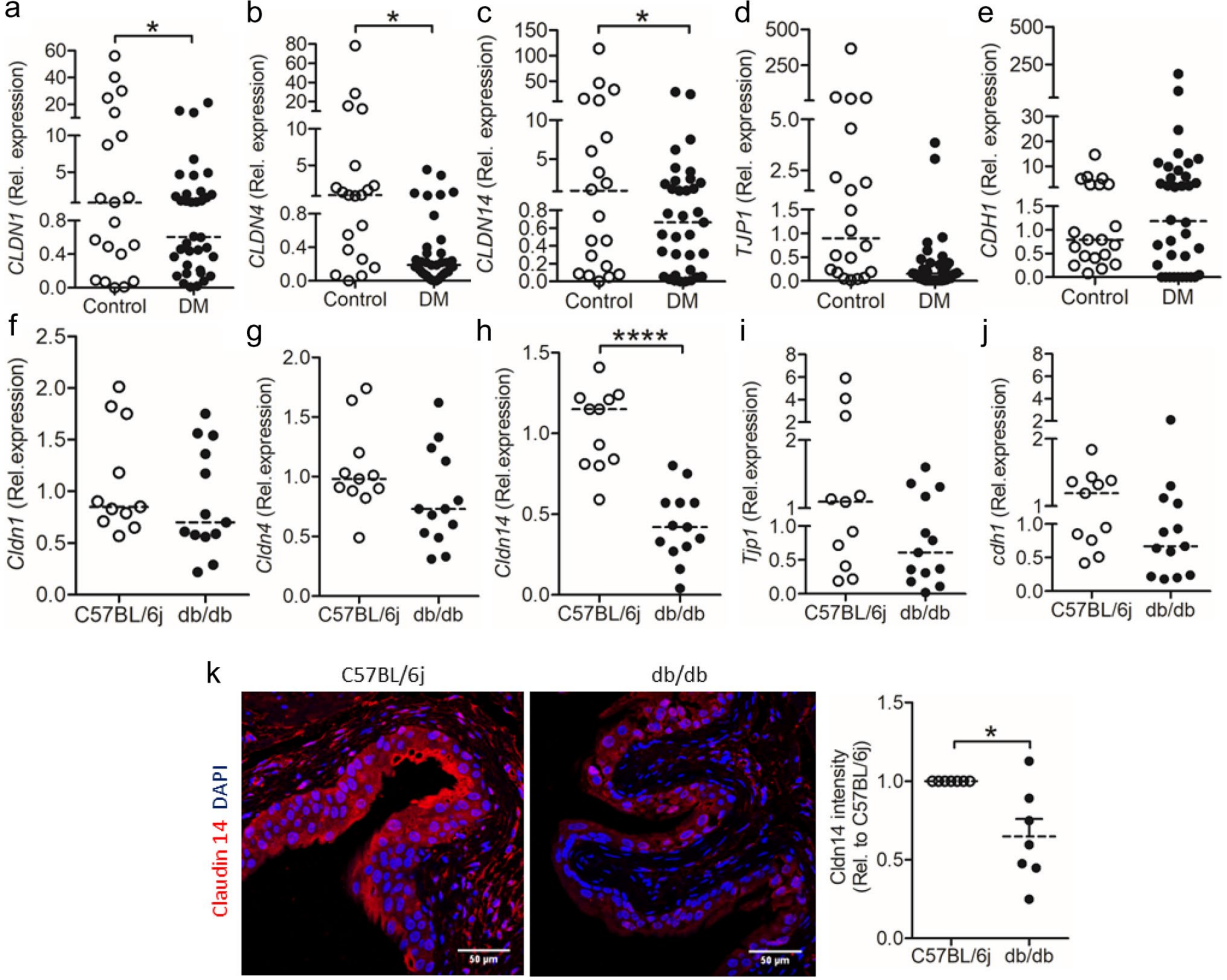
Diabetes significantly downregulates expression of claudin 14 in uroepithelial cells in humans and mice. Expression of human **a** claudin 1 (*CLDN1)*, **b** claudin 4 (*CLDN4)*, **c** claudin 14 (*CLDN14)*, **d** tight junction protein 1 (*TJP1)*, and **e** cadherin (*CDH1*) in urine exfoliated cells from patients with diabetes (DM, *n* = 36) and non-diabetic individuals (*n* = 20), unpaired, two-tailed *t*-test. Expression of mouse **f** claudin 1 (*Cldn1*), **g** claudin 4 (*Cldn4*), **h** claudin 14 (*Cldn14*), **i** tight junction protein 1 (*Tjp1*), and **j** E cadherin (*Cdh1*) mRNA in diabetic (db/db, *n* = 11) and non-diabetic (C57BL/6j, *n* = 7) mice, unpaired, two-tailed *t*-test.** k** Claudin 14 protein expression in diabetic (db/db, *n* = 7) and non-diabetic (C57BL/6j, *n* = 7) mice, paired *t*-test. For in vivo analysis, median is shown. *p < 0.05 and ****p < 0.0001

Tight junction proteins are integral components of the epithelium and responsible for cell to cell adhesion and control paracellular permeability. To investigate the possible impact of diabetes on cell junction proteins in the urinary bladder, exfoliated uroepithelial cells from the urine of patients with diabetes, median HbA1c of 53.5 mmol/mol, were analyzed (Mohanty et al. 2022). Previously, we reported that diabetes downregulates the antimicrobial peptide psoriasin impacting the expression of tight junction protein, occludin (Mohanty et al. 2022). Psoriasin is also known to regulate claudin family proteins like claudin 1, 4, and 14 in skin epithelial cells (Hattori et al. 2014). Since mRNA levels of urine exfoliated cells highlight the expression pattern of tight junction proteins in the bladder tissue, we chose to study proteins of the claudin family. Interestingly, we observed lower mRNA expression of claudin 1 (*CLDN1*, Fig. 1A), claudin 4 (*CLDN4*, Fig. 1B), and claudin 14 (*CLDN14*, Fig. 1C) in patients with diabetes, and clear downregulation in the expression of tight junction protein 1 (*TJP1*, Fig. 1D) although it did not reach significance. No difference in the expression of E cadherin (*CDH1*, Fig. 1E) was observed when compared to non-diabetic controls.

To confirm our results, urinary bladders from mice with hereditary diabetes, db/db, were analyzed. A significant downregulation in *Cldn14* mRNA (Fig. 1H) and a clear trend of downregulation in mRNA of *Cldn1* (Fig. 1F), *Cldn4* (Fig. 1G), *Tjp1* (Fig. 1I), and *cdh1* (Fig. 1J) were observed even though they did not reach significance when compared to non-diabetic, C57BL/6j mice. Interestingly, only the mRNA expression of claudin 14 was consistent in human urine exfoliated cells and mouse bladders. In line with this, on the protein level, we detected a significant reduction in claudin 14 in the superficial layer of db/db bladder when compared to C57BL/6j mice (Fig. 1K). To further improve to explore other known and yet unknown tight junction proteins which might help understand the role of the barrier function, we believe that non-diabetic, db/+ control mice will open new additional possibilities.

**Fig. 2:**
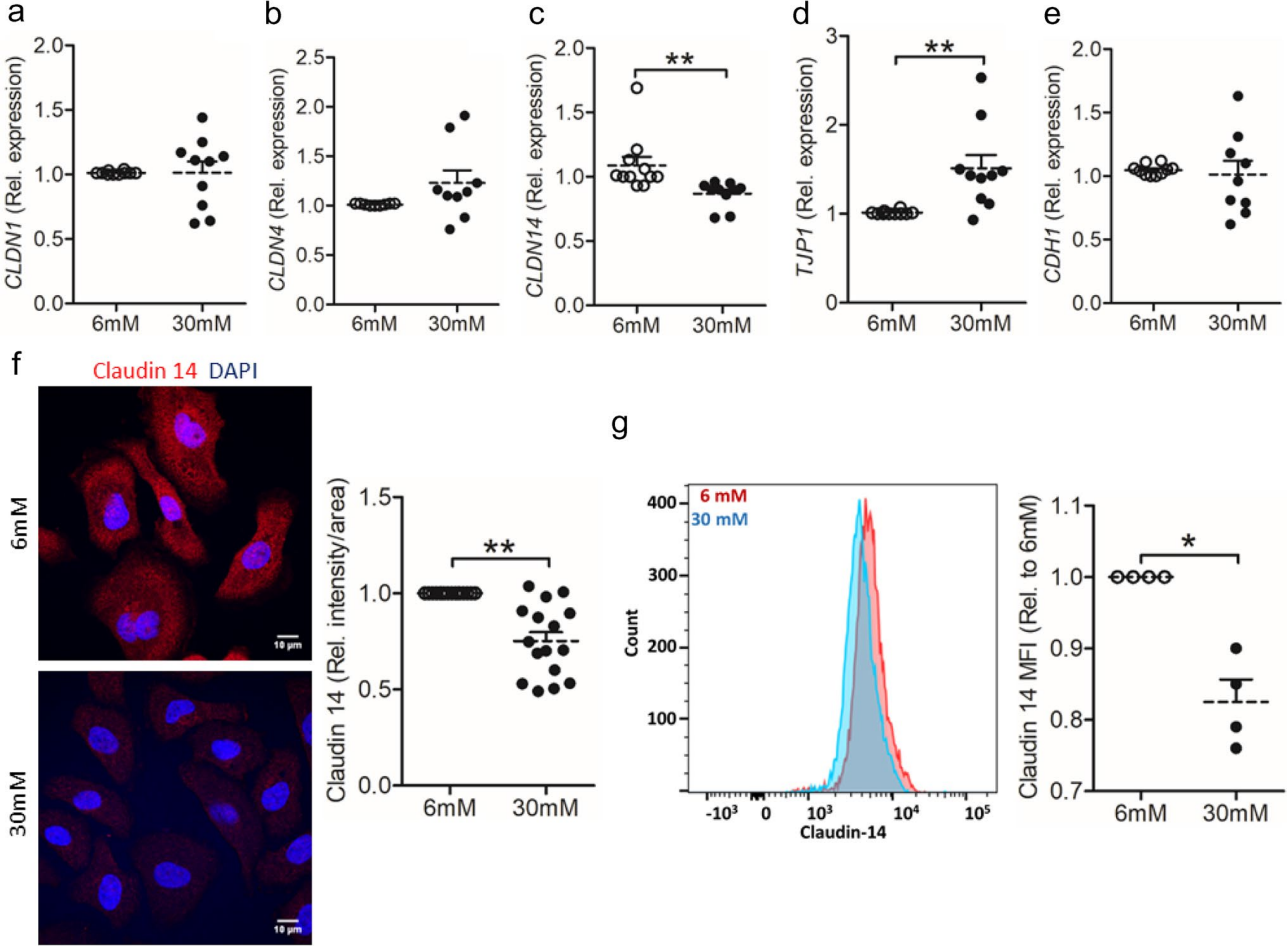
High glucose significantly compromises the expression of claudin 14 in human uroepithelial cells. Expression of human **a** claudin 1 (*CLDN1*), **b** claudin 4 (*CLDN4*), **c** claudin 14 (*CLDN14*), **d** tight junction protein 1 (*TJP1*), and **e** E cadherin (*CDH1*) mRNA levels in TERT-NHUC uroepithelial cells cultured with normal (6 mM) and high (30 mM) glucose for 24 h, unpaired, two-tailed *t*-test. **f** Claudin 14 protein expression in high glucose-treated TERT-NHUC uroepithelial cells, paired *t*-test. **g** Analysis of claudin 14 (MFI, flow cytometry) expression in TERT-NHUC cells after 36 h glucose treatment, paired *t*-test. In vitro experiment was performed in duplicate/triplicate with at least 3 independent experiments and presented as mean ± SEM. Statistical outliers defined by Grubbs’ test were excluded. *p < 0.05 and **p < 0.01

The reduction of claudin 14 in db/db bladder prompted us to further investigate, if human uroepithelial cells, TERT-NHUC, were similarly influenced. High glucose (30 mM) resulted in lower mRNA expression of *CLDN14* overall (Fig. 2C) and in the plasma membrane (Supplementary Fig. 1), with increased expression observed in *TJP1* (Fig. 2D) whereas no difference was observed in the mRNA expression of *CLDN1* (Fig. 2A), *CLDN4* (Fig. 2B), and *CDH1* (Fig. 2E) when compared to low glucose (6 mM) condition. A significant difference of *TJP1* at the mRNA level between low and high glucose-treated TERT-NHUC cells was observed. However, microscopic analysis of TJP1 did not show any difference in the expression of TJP1 in 30 mM after 36 h of treatment (Supplementary Fig. 2). Furthermore, microscopic (Fig. 2F) and flowcytometric analysis (Fig. 2G) of human uroepithelial TERT-NHUC cells in vitro also revealed decreased claudin 14 protein levels in high compared to low glucose-treated cells.

Our data highlight a strong impact of high glucose on the expression of claudin 14. High glucose is known to reduce the expression of other cell junction proteins like occludin (Mohanty et al. 2022), connexin 43 (Sato et al. 2002), and claudins 2 and 5 (Rosas-Martínez et al. 2021). Our observation in diabetic, db/db, mice is of importance in the context of UTI as these mice are known for compromised intestinal barrier with higher risk of enteric infection (Thaiss et al. 2018).

**Fig. 3:**
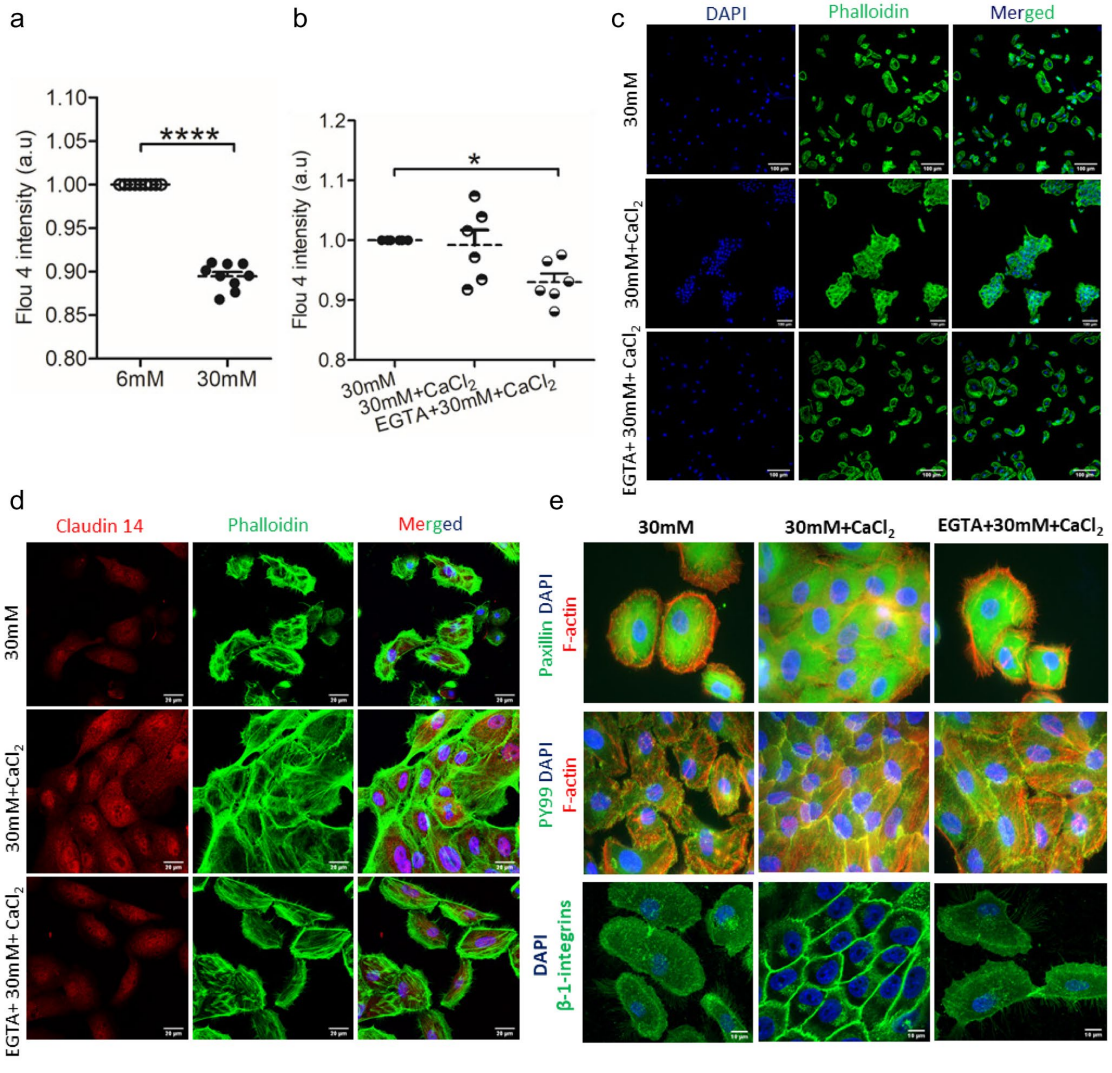
Extracellular calcium modulates claudin 14 expression along with β-1 integrins and focal adhesion kinase in human uroepithelial cells. Intracellular calcium levels in TERT-NHUC cells **a** under normal (6 mM) *vs* high (30 mM) glucose, paired *t*-test, and **b** extracellular supplementation of calcium with/without in calcium chelated (EGTA-treated) high glucose cells. One-way ANOVA. Effect of calcium on **c** cell cluster formation, shown via phalloidin and DAPI staining and **d** claudin 14 expression in calcium with/without EGTA-treated high glucose TERT-NHUC cells. **e** Impact of extracellular calcium on the expression of focal adhesion kinase markers like paxillin and phosphotyrosine (PY99); and β-1 intergrins in high glucose and calcium with/without EGTA-treated TERT-NHUC cells. In vitro experiment was performed in duplicate/triplicate with at least 3 independent experiments and presented as mean ± SEM. *p < 0.05 and ****p < 0.0001

However, how glucose specifically affects claudin 14 remains unclear. In the context of the urinary tract this is of particular interest as claudin 14 is responsible for the renal Ca^2+^ transport (Gong et al. 2012). Therefore, we next analyzed the influence of high glucose on the intracellular calcium levels. High glucose treatment in TERT-NHUC resulted in lower intracellular calcium levels (Fig. 3A), which could be due to decreased claudin 14. The role of calcium (1 mM) has been studied to analyze the uroepithelial barrier function (Truschel et al. 1999). Furthermore, we supplemented with a non-cytotoxic dose of 1 mM calcium (Supplementary Fig. 3) in high glucose-treated cells which did not increase the intracellular calcium load. In contrast, extracellular calcium chelation using EGTA (1 mM) resulted in lower intracellular calcium, thereby indicating a reduction of basal calcium levels which was not recovered with supplementation with external calcium (Fig. 3B).

Extracellular increase in calcium levels markedly influenced the morphology of TERT-NHUC cells in vitro, with a significant migration of cells to form tightly packed clusters (Fig. 3C). This observation was completely inhibited in the presence of EGTA. To our surprise, in TERT-NHUC cells high glucose increased the expression of *TJPI* mRNA, which is known to interact with the cytoskeleton and is crucial for mechano-sensing and mechano-transduction (Citi 2019). Claudin 14 interacts with TJP1; therefore, we hypothesized that calcium mediated alteration in TERT-NHUC cells could influence claudin 14 expression. Interestingly, we observed increased expression of claudin 14 in calcium and high glucose-treated cells when compared to EGTA and high glucose-treated cells only (Fig. 3D). However, we cannot rule out the role of other claudin family proteins like claudin 2, 16, and 19 in reabsorption of calcium.

Local rise in calcium increases the residency of focal adhesion kinase (FAK) at focal adhesions (Giannone et al. 2004). Further to confirm calcium-mediated cellular migration, we analyzed focal adhesion disassembly measured by altered paxillin localization (Fig. 3E). We also analyzed relocalization of focal adhesion components to cell to cell junctions measured by altered β-1-integrin and tyrosine phosphorylated proteins, possibly by the activity of FAK. During calcium treatment, phosphotyrosine signal accumulated at cell to cell junctions, and focal adhesions (Fig. 3E). FAK senses extracellular stimuli and initiates the signaling cascades that promote cell migration (Zhao et al. 2016). We observed increased β-1-integrin expression in calcium but not EGTA and high glucose-treated cells (Fig. 3E). The observation in high glucose-treated uroepithelial cells is of interest as FAK regulates fibroblast migration using β-1-integrins (Zhao et al. 2016). In addition, β-1-integrins regulate paracellular permeability of kidney proximal tubule cells (Elias et al. 2014) where claudin proteins play a vital role. It is also of prime importance that induction of FAK via calcium supplementation could have a detrimental effect as FAK is also responsible for bladder bacterial invasion (Martinez et al. 2000). Therefore, a detailed investigation is required in the future before possible clinical use.

## Conclusions

High glucose decreases the expression of the tight junction protein claudin 14 in vivo and in vitro in the context of the urothelium. This could be a potential reason of increased risk of infections in the urinary bladder. Further, calcium supplementation in vitro reverts this effect. However, a limitation of the study is that we were not able to include clinical data of persons with diabetes after calcium supplementation and to explore the calcium effect on infection. Future studies may investigate calcium levels and the necessity to maintain adequate levels in patients with diabetes as a prophylactic treatment against UTI.

## Supplementary Information

Below is the link to the electronic supplementary material.

Supplementary file1 (DOCX 393 KB)

## Data Availability

No datasets were generated or analysed during the current study.
