# Supplementary material for: Diabetes compromises tight junction protein claudin 14 in the urinary bladder
Source: Cell Tissue Res. 2024 Aug 20;398(1):27–33. doi: 10.1007/s00441-024-03908-4 (PMC11424655; doi:10.1007/s00441-024-03908-4)
Supplement: Supplementary file 1 — Supplementary file1 (DOCX 393 KB) [file 441_2024_3908_MOESM1_ESM.docx]

***Supplementary file***

**Diabetes compromises tight junction protein claudin 14 in the urinary bladder**

Soumitra Mohanty *^, # 1,2^, John Kerr White^1,2^, Andrea Scheffschick^3^, Berenice Fischer^3^, Anuj Pathak^2^, Jonas Tovi ^4^, Claes-Göran Östenson^5^, Pontus Aspenström^6^, Hanna Brauner^3,7,8^, Annelie Brauner *^1,2^.

^1^ Department of Microbiology, Tumor and Cell Biology, Karolinska Institutet, Stockholm, Sweden.

^2^ Division of Clinical Microbiology, Karolinska University Hospital, Stockholm, Sweden

^3^ Division of Dermatology and Venereology, Department of Medicine, Solna, Stockholm, Sweden
^4^ Capio Health Care Center, Solna, Sweden

^5^ Department of Molecular Medicine and Surgery, Karolinska Institutet, Stockholm, Sweden

^6^ Rudbeck Laboratory, Department of Immunology, Genetics and Pathology (IGP), Uppsala University, Uppsala, Sweden

^7^ Division of Dermatology and Venereology, Karolinska University Hospital, Stockholm, Sweden.

^8^ Center for Molecular Medicine, Karolinska Institutet

* Shared corresponding authors:

Soumitra Mohanty and Annelie Brauner. E-mail: [sm14@nibmg.ac.in](mailto:sm14@nibmg.ac.in) , [Annelie.Brauner@ki.se](mailto:Annelie.Brauner@ki.se)

^#^ Present address: Biotechnology Research and Innovation Council-National Institute of Biomedical Genomics (BRIC-NIBMG), Kalyani, India

| **Gene name** | **Sequence (5’-3’)** |
| --- | --- |
| Human *CLDN1* (Forward) | TCCACTGAACAAAACCTACGC |
| Human *CLDN1* (Reverse) | TGAAAAGCAACACCAAAACG |
| Mouse *Cldn1* (Forward) | CTGGGTTTCATCCTGGCTTC |
| Mouse *Cldn1* (Reverse) | TTGATGGGGGTCAAGGGGTC |
| Human *CLDN4* (Forward) | GGCTGCTTTGCTGCAACTGTC |
| Human *CLDN4* (Reverse) | GAGCCGTGGCACCTTACACG |
| Mouse *Cldn4* (Forward) | GGGAATCTCCTTGGCAGTC |
| Mouse *Cldn4* (Reverse) | GGCGAGCATCGAGTCGTA |
| Human *CLDN14* (Forward) | TGTACCTGGGCTTCATCTCC |
| Human *CLDN14* (Reverse) | CCTCGCATTCACATTATTTCC |
| Mouse *Cldn14* (Forward) | CACACCCGCCAAGACCACCT |
| Mouse *Cldn14* (Reverse) | AGGTACAGGGCCTGGCCGAT |
| Human *TJP1* (Forward) | CGGTCCTCTGAGCCTGTAAG |
| Human *TJP1* (Reverse) | GGATCTACATGCGACGACAA |
| Mouse *Tjp1* (Forward) | CGAGTTGCAATGGTTAACGGA |
| Mouse *Tjp 1* (Reverse) | TCAGGATCAGGACGACTTACTGG |
| Human *CDH1* | Hs01023895_ml |
| Mouse *Cdh1* (Forward) | GGTTTTCTACAGCATCACCG |
| Mouse *Cdh1* (Reverse) | GCTTCCCCATTTGATGACAC |
| Human *ACT B* (Forward) | AAGAGAGGCATCCTCACCCT |
| Human *ACT B* (Reverse) | TACATCGCTGGGGTGTTG |
| Mouse *Act b* (Forward) | CTGTCCCTGTATGCCTCTG |
| Mouse *Act b* (Reverse) | ATGTCACGCACGATTTCC |
| Human *GAPDH* | 4326317E |

**Table 1:** List of primers and probes used in this study.

| **Protein name** | **Primary antibody** | **Primary antibody dilution** | **Secondary**  **antibody** | **Secondary**  **antibody dilution** |
| --- | --- | --- | --- | --- |
| Claudin 14 | Abcam, ab19035 | 1:200 (IF)  1:100 (Flow) | Alexa 594, A21468  Alexa 488, A21467 | 1:400 (IF and Flow) |
| Paxillin  Mouse monoclonal | BD Transduction Laboratories  #610051 | 1:200 (IF) | Alexa 488, A21202 | 1:500 (IF) |
| Phosphotyrosine (PY99) mouse monoclonal | Santa Cruz, SC-7020 | 1:100 (IF) | Alexa 488, 21202 | 1:500 (IF) |
| β-1 integrins | Santacruz,  SC-73645 | 1:200 (IF) | Alexa 488, A11059 | 1:400 (IF) |
| F-actin | Sigma-Aldrich, TRITC-phalloidin P1951 | 1:350 (IF) |  |  |
| Phalloidin | Invitrogen,  A12379 | 1:1000 (IF) |  |  |

**Table 2:** List of antibodies used in this study.

**Supplementary fig.1**


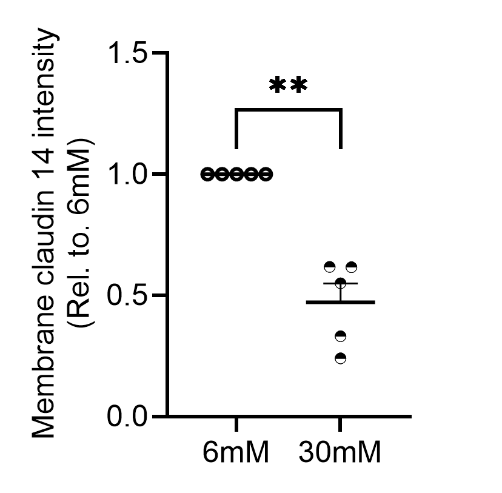


**Supplementary fig. 1 Expression of claudin 14.** Intensity of claudin 14 protein expression in the plasma membrane of high glucose (30mM) in comparison to normal glucose (6mM) treated cells, TERT-NHUC was quantified using image J. Paired, t-test.**≤0.01.

**Supplementary fig.2**

**
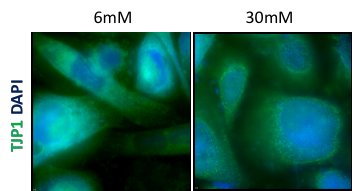
**

**Supplementary fig. 2 Expression of TJP1.** Intensity of TJP1protein expression in high glucose (30mM) in comparison to normal glucose (6mM) treated cells, TERT-NHUC.

**Supplementary fig. 3**


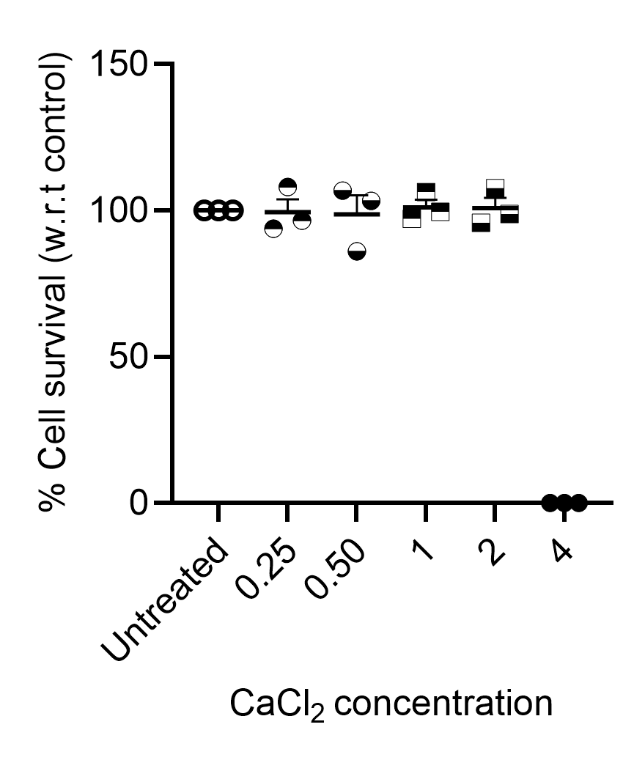


**Supplementary fig. 3:** **Cytotoxicity of CaCl_2_**_._ Cytotoxicity of CaCl_2_ with different concentrations was performed in high glucose (30mM) treated human uroepithelial cells, TERT-NHUC post 24 h of treatment using trypan blue staining.
